# Serum γ-glutamyltransferase and uric acid levels are associated with impaired fasting glucose in adults from Inner Mongolia, China

**DOI:** 10.1186/1471-2458-13-294

**Published:** 2013-04-03

**Authors:** Jie Wu, Ling Qiu, Wen-hua Yan, Xin-qi Cheng, Wei Wu, Xiu-zhi Guo, Hai-tao Ding, Hui-juan Han, Shao-mei Han, Guang-jin Zhu

**Affiliations:** 1 Department of Clinical Laboratory, Peking Union Medical College Hospital, Chinese Academy of Medical Sciences & Peking Union Medical College, Beijing, 100730, China; 2 Department of Endocrinology, Chinese PLA General Hospital, Beijing, 100853, China; 3 Department of Clinical Laboratory, Inner Mongolian People’s Hospital, Hohhot, 010017, China; 4 Department of Pathophysiology, Institute of Basic Medical Sciences, Chinese Academy of Medical Sciences & Peking Union Medical College, Beijing, 100005, China

**Keywords:** γ-Glutamyltransferase, Uric acid, Prediabetes, Impaired fasting glucose

## Abstract

**Background:**

Serum γ-glutamyltransferase (GGT) and uric acid (UA) levels are elevated in patients with diabetes or cardiovascular disease. Prediabetes, characterized by impaired glucose tolerance, is an important risk factor for overt diabetes as well as cardiovascular disease. Therefore, the aim of this study was to explore the relationship between GGT, UA and prediabetes in a Chinese population, and provide a scientific basis for the early prevention and treatment of diabetes.

**Methods:**

We performed a cross-sectional population-based study in a cohort of 2694 subjects (1211 men and 1483 women, aged 35–86 years). Questionnaires and physical examinations were performed using standardized procedures. Fasting blood was collected to measure glucose and other biochemical parameters. The subjects were divided into two groups with either normal fasting glucose (NFG) or impaired fasting glucose (IFG), according to international diagnostic criteria. Logistic regression analysis was performed to estimate odds ratios (OR) and 95% confidence intervals.

**Results:**

Compared with the NFG group, the IFG group had significantly higher blood pressure but lower high-density lipoprotein–cholesterol in women. Body mass index, waist circumference, triglyceride, glucose, GGT, and UA levels were significantly higher in males and females in the IFG group than those in the NFG group. Logistic regression analysis revealed that the OR for prediabetes increased with increasing serum GGT quartiles and UA quartiles. GGT and UA were positively associated with prediabetes in men and women, independent of age, ethnicity, smoking, alcohol consumption, blood pressure, physical labor, and other confounders.

**Conclusions:**

We found that serum GGT and UA levels were positively associated with prediabetes in men and women living in areas inhabited by Chinese ethnic minorities. As elevated GGT and UA levels were associated with significantly increased risk of prediabetes, they may be used as sensitive biological markers of prediabetes.

## Background

Serum γ-glutamyltransferase (GGT), which is mainly derived from the liver, is a sensitive marker of liver cell damage and oxidative stress. Uric acid (UA) is the final oxidation product of human purine metabolism and is used clinically as a marker of inflammation and metabolic disease. Recent studies have revealed that elevated serum GGT levels and hyperuricemia are closely associated with diabetes and cardiovascular disease [1-3]. Therefore, recent studies that examined the associations of serum GGT and UA levels with clinically defined blood glucose categories corresponding to early stages of diabetes have received considerable attention in terms of diabetes prevention.

Prediabetes is an important risk factor for the development of overt diabetes as well as cardiovascular disease [4,5], and is defined as impaired fasting glucose (IFG) and/or glucose tolerance (IGT). The American Diabetes Association defines IFG as fasting serum glucose concentrations of 5.6–6.9 mmol/L (100–125 mg/dL) and IGT as serum glucose concentrations of 7.8–11.1 mmol/L (140–199 mg/dL) at 2 h after a 75 g oral glucose load [6]. IFG is associated with insulin resistance and with a greater conversion from prediabetes to overt diabetes (approximately 24% within 3 years) compared with IGT [7]. Nationwide surveys conducted in the United States revealed that prediabetes was very prevalent among adolescents [8,9]. In a recent population-based study involving >45,000 people in China, the prevalence of prediabetes was 15.5% [10].

In our previous study, we observed higher GGT and UA levels in subjects with coexisting prehypertension and prediabetes, which suggests that GGT and UA are associated with prediabetes [11]. Similarly, results of several surveys conducted in the United States and other countries implicated GGT and/or UA in the development of diabetes [12-15]. The associations of serum GGT and UA levels with plasma glucose levels were reported recently among Chinese adults in Qingdao, a coastal city of China [16,17]. However, China is a multi-ethnic country with marked regional differences, and little is known about the associations of serum GGT and UA levels with prediabetes among individuals living in areas inhabited by Chinese ethnic minorities. Therefore, in this study, we enrolled a representative cohort of subjects living in Inner Mongolian Autonomous Region using a random, multistage cluster sampling scheme. The enrolled subjects completed a questionnaire, and underwent physical examinations and biochemical tests. We examined the associations of GGT and UA with IFG as a marker of prediabetes, and determined the potential risk factors for prediabetes in this population.

## Methods

### Study population

A population-based, cross-sectional survey of the Chinese Physiological Constant and Health Condition was conducted between 2009 and 2010. A representative sample of the general Chinese population, aged 35–86 years, in two urban and two rural areas of the Inner Mongolian Autonomous Region was identified using a random, multistage cluster-sampling method. Written informed consent was obtained from each participant before data collection. The protocol was approved by the Institutional Review Board of the Institute of Basic Medical Sciences, Chinese Academy of Medical Sciences. Trained medical personnel collected information on risk factors via questionnaires, and obtaining anthropometric measurements and blood samples for biochemical assessments.

### Exclusion criteria

Participants with known systemic diseases, including diabetes mellitus, hypertension, cardiovascular disease, renal disease, gastrointestinal disease, pulmonary disease, or cancer were excluded. Participants taking any drugs known to affect carbohydrate or lipid metabolism were also excluded. After applying these criteria, 2694 participants were eligible for this study and were analyzed.

### Data collection

All of the participants completed a standard questionnaire, which included demographic characteristics (e.g., age, sex, and ethnicity), physical condition, lifestyle habits and customs (e.g., smoking status, alcohol consumption, and manual labor), past medical history, and lifestyle risk factors. Manual labor was classified as light physical (i.e., students and white-collar workers), moderate physical (i.e., staff and teachers), and heavy physical (i.e. farmers and blue-collar workers). Body weight was measured to the nearest 0.1 kg using a calibrated beam scale and height was measured barefoot three times using a wall-mounted stadiometer (Seca, Hamburg, Germany) to the nearest 0.1 cm. Body mass index (BMI) was calculated as body weight (in kilograms) divided by height (in meters squared). Waist circumference (WC) was measured midway between the lower rib margin and the iliac crest at the end of a gentle expiration. Blood pressure (BP) was measured using OMRON HEM-7000 electronic sphygmomanometer (OMRON HealthCare, Kyoto, Japan) after the participant had rested for ≥10 min. The participant’s arm was placed at the level of the heart and the mean of three BP measurements was used.

### Laboratory measurements

All procedures were performed after a 12-h overnight fast. Blood was drawn from the antecubital vein of the right arm. Serum GGT, UA, fasting blood glucose (FBG), and other biochemical tests were assayed with an Olympus AU2700 Automatic Biochemical Analyzer (Olympus, Tokyo, Japan). The participants were classified according to FBG as having normal FBG (<5.6 mmol/L; NFG) or IFG (FBG 5.6–6.9 mmol/L) according to the American Diabetes Association criteria [6]. Lipid profiles, including total cholesterol (TC), triglycerides (TG), high-density lipoprotein–cholesterol (HDL-C), and low-density lipoprotein–cholesterol (LDL-C) were also measured. The biochemical laboratories participating in the survey adhered to the same internal quality control program that was standardized by Peking Union Medical College Hospital. The laboratory reference ranges for GGT and UA are 0–50 U/L and 104–444 U/L, respectively.

### Statistical analysis

Data were analyzed using SPSS 13.0 software (SPSS Inc., Chicago, IL, USA). Normally distributed continuous variables are presented as means ± standard deviation (SD). All categorical data are presented as percentages. Comparisons between groups for normally distributed variables were made using an analysis of covariance F-test. GGT, TG, aspartate aminotransferase (AST), alanine aminotransferase (ALT), alkaline phosphatase (ALP), and creatine kinase (CK) levels showed skewed distributions and are presented as medians (interquartile range). These variables were compared between groups using the Wilcoxon rank sum test. Correlation analysis and linear regression were used to determine the associations between biochemical indicators and fasting blood glucose. The participants were then divided into quartiles of sex-specific serum GGT and UA levels. Multivariate logistic regression was used to calculate the odds ratio (OR) and 95% confidence intervals (CI), as well as the corresponding *P*-values and effects of each categorical variable. All statistical tests were two-tailed, and considered significant at *P* < 0.05.

## Results

### Clinical characteristics of the IFG and NFG groups stratified by sex

As shown in Table 1, there was no significant difference in mean age between the IFG and NFG groups for men, but the IFG group was older than the NFG group for women. The prevalence of IFG was higher in men (27.5%) than in women (17.5%). Among men, the proportions of smokers and drinker were greater in the IGF group than in the NFG group. Among women, the IFG group had significantly higher systolic and diastolic blood pressures, but lower HDL-C levels compared with the NFG group (*P* < 0.05). Regardless of sex, the prevalence of prediabetes was higher in Han than in Mongolian participants. BMI, WC, TG, GLU, GGT, CK, and UA were significantly greater in the IFG group than in the NFG group (*P* < 0.05).

**Table 1:** Baseline characteristics of participants according to FBG levels

	**Male**		**Female**	
	**NFG**	**IFG**	**NFG**	**IFG**
n (%)	878 (72.5%)	333 (27.5%)	1224 (82.5%)	259 (17.5%)
Age (years)	52.97 ± 11.68	53.39 ± 11.60	50.62 ± 10.20	54.39 ± 10.87*
Ethnic group (%)				
Han	624 (71.1%)	266 (79.9%)	801 (65.4%)	219 (84.5%)
Mongolian	235 (26.8%)	56 (16.8%)	398 (32.5%)	35 (13.5%)
Other	19 (2.1%)	11 (3.3%)	26 (2.1%)	5 (2%)
Smoking (%)	387 (44.1%)	155 (46.5%)	76 (6.2%)	16 (6.2%)
Drinking (%)	449 (51.1%)	188 (56.5%)	64 (5.2%)	14 (5.4%)
Manual labor (%)				
Light	528 (60.1%)	187 (56.2%)	771 (63.0%)	174 (67.2%)
Moderate	140 (15.9%)	64 (19.2%)	27 (22.1%)	45 (17.4%)
Heavy	123 (14.0%)	45 (13.5%)	77 (6.3%)	17 (6.6%)
BMI (kg/m^2^)	25.2 ± 3.6	26.1 ± 3.6*	24.8 ± 3.5	25.7 ± 3.4*
WC (cm)	88.2 ± 10.0	90.0 ± 9.5*	81.1 ± 9.4	84.8 ± 9.0*
SBP (mmHg)	129.1 ± 15.8	130.8 ± 14.6	123.3 ± 15.5	127.5 ± 16.3*
DBP (mmHg)	83.2 ± 10.6	84.4 ± 10.6	79.8 ± 9.6	81.6 ± 9.9*
TC (mmol/L)	5.16 ± 1.05	5.37 ± 1.07*	5.16 ± 0.99	5.27 ± 1.08
TG (mmol/L)	1.50 (1.08–2.32)	1.98 (1.31–2.95)*	1.32 (1.00–1.79)	1.72 (1.14–2.50)*
HDL-C (mmol/L)	1.32 ± 0.29	1.29 ± 0.27	1.44 ± 0.29	1.37 ± 0.29*
LDL-C (mmol/L)	2.85 ± 0.85	2.93 ± 0.87	2.80 ± 0.81	2.86 ± 0.84
GLU (mmol/L)	5.03 ± 0.38	6.03 ± 0.38*	4.98 ± 0.34	6.00 ± 0.33*
AST (U/L)	24.5 (20.8–29.8)	24.7 (21.2–30.1)	22.1 (18.9–26.1)	23.9 (20.1–28.2)
ALT (U/L)	24.1 (17.9–34.3)	26.7 (19.0–40.0)	17.5 (13.6–24.0)	21.5 (15.8–29.7)*
GGT (U/L)	32.4 (21.5–32.4)	40.3 (26.4–63.8)*	19.1 (14.0–28.2)	24.2 (17.3–39.4)*
ALP (U/L)	81.7 (69.2–94.5)	81.7 (68.0–95.1)	76.3 (62.2–97.2)	84.0 (68.0–100.1)*
CK (U/L)	98.9 (76.2–135.1)	103.0 (82.6–139.1)*	75.4 (60.0–100.0)	80.7 (61.5–108.2)*
UA (μmmol/L)	347.1 ± 78.8	365.6 ± 83.3*	262.1 ± 64.6	297.5 ± 69.5*

^*1*^ Abbreviations: *FBG*, Fasting blood glucose; *NFG*, Normal fasting glucose; *IFG*, Impaired fasting glucose; *BMI*, Body mass index; *WC*, Waist circumference; *SBP*, Systolic blood pressure; *DBP*, Diastolic blood pressure; *TC*, Total cholesterol; *TG*, Triglyceride; *HDL*-*C*, High density lipoprotein cholesterol; *LDL*-*C*, Low density lipoprotein cholesterol; *GLU*, Glucose; *AST*, Aspartate aminotransferase; *ALT*, Alanine aminotransferase; *GGT*, γ-Glutamyltransferase; *ALP*, Alkaline phosphatase; *CK*, Creatine kinase; *UA*, Uric acid.

^*2*^ Data are presented as n (%), mean ± standard deviation (SD); TG, AST, ALT, GGT, ALP, and CK were reported as medians (interquartile range).

^*3*^ *P < 0.05 for IFG vs. NFG in the same sex.

### Correlations between liver enzymes, UA, and FBG levels

We next explored the correlations of GGT, ALP, CK and UA with FBG levels using correlation and linear regression analyses (Table 2). Because of their skewed distributions, serum GGT, ALP, and CK were log-transformed before the analyses. The results showed that GGT and UA levels were significantly and positively associated with FBG levels in both sexes. ALP and CK were associated with FBG in females, and the association disappeared after adjusting for age and other factors using logistic regression analysis. In men, the standardized coefficients of GGT and UA were 0.144 and 0.133, with R^2^ values of 2.1% and 1.8%, respectively. In women, the standardized coefficients of GGT and UA were 0.170 and 0.220, with R^2^ values of 2.9% and 4.8%, respectively. These coefficients and R^2^ values indicate that FBG levels were also influenced by other factors. Therefore we need to exclude confounding factors (such as age and sex) to further confirm the correlations.

**Table 2:** Correlations between GGT, ALP, CK, and UA levels and FBG levels

	**FBG**		
	**Standard β coefficient**	**R**^**2**^	***P*values**
Males (n = 1211)			
GGT	0.144	0.021	< 0.001
ALP	0.008	0.000	0.772
CK	0.036	0.001	0.215
UA	0.133	0.018	< 0.001
Females (n = 1483)			
GGT	0.170	0.029	< 0.001
ALP	0.110	0.012	< 0.001
CK	0.053	0.003	0.043
UA	0.220	0.048	< 0.001

^*1*^ Abbreviations: *GGT*, γ-Glutamyltransferase; *ALP*, Alkaline phosphatase; *CK*, Creatine kinase; *UA*, Uric acid; *FBG*, Fasting blood glucose.

^*2*^ Correlation and linear regression analyses were used to estimate the correlations between each variable and FBG.

### Non-conditional logistic regression analysis of the associations of serum GGT and UA quartiles with IFG

Table 3 shows sex-specific associations of increasing GGT and UA levels with IFG. In these analyses, IFG (no = 0, yes = 1) was used as the dependent variable, and quartiles of GGT and UA levels were included as independent variables and analyzed in three risk models. The OR and 95% CI for IFG were calculated for each sex-specific GGT/UA quartile, with the lowest quartile as the reference category, in multivariate logistic regression models. Among men and women, GGT and UA quartiles were associated with IFG in all three models after accounting for the effects of confounding factors. The linear trends in these associations were also statistically significant.

**Table 3:** Associations of sex-specific serum GGT and UA quartiles with IFG

	**Subgroup**	**IFG**	**OR (95% CI)**		
	**(n)**	**n (%)**	**Model 1**	**Model 2**	**Model 3**
**Males**					
GGT					
Q1 (<23 U/L)	312	59 (18.9%)	1 (ref)	1 (ref)	1 (ref)
Q2 (23–35 U/L)	305	78 (25.6%)	1.62 (1.06–2.50)	1.50 (0.97–2.31)	1.48 (0.94–2.29)
Q3 (35–57 U/L)	291	94 (32.3%)	0.91 (0.62–1.35)	2.08 (1.32–3.25)	1.99 (1.26–3.15)
Q4 (>57 U/L)	303	102 (33.7%)	1.01 (0.68–1.51)	2.14 (1.33–3.45)	1.90 (1.16–3.12)
*P* for trend			0.000	0.001	0.007
UA					
Q1 (<296 U/L)	225	71 (31.6%)	1 (ref)	1 (ref)	1 (ref)
Q2 (296–344 U/L)	236	67 (28.4%)	0.93 (0.61–1.40)	0.84 (0.55–1.27)	0.84 (0.55–1.28)
Q3 (344–402 U/L)	221	87 (39.4%)	1.43 (0.96–2.13)	1.23 (0.82–1.85)	1.19 (0.79–1.81)
Q4 (>402 U/L)	194	107 (55.2%)	1.91 (1.28–2.84)	1.52 (1.00–2.31)	1.47 (0.96–2.25)
*P* for trend			0.000	0.013	0.027
**Females**					
GGT					
Q1 (<15 U/L)	409	38 (9.3%)	1 (ref)	1 (ref)	1 (ref)
Q2 (15 ~ 20 U/L)	338	49 (14.5%)	1.76 (1.06–2.94)	1.65 (0.99–2.78)	1.58 (0.94–2.67)
Q3 (20 ~ 30 U/L)	366	74 (20.2%)	2.69 (1.63–4.45)	2.44 (1.46–4.08)	2.26 (1.33–3.83)
Q4 (>30 U/L)	370	98 (26.5%)	3.52 (2.17–5.72)	3.19 (1.93–5.27)	2.86 (1.70–4.82)
*P* for trend			0.000	0.000	0.000
UA					
Q1 (<220 U/L)	330	36 (10.9%)	1 (ref)	1 (ref)	1 (ref)
Q2 (220–262 U/L)	325	42 (12.9%)	1.16 (0.71–1.90)	1.09 (0.66–1.80)	1.08 (0.65–1.78)
Q3 (262–310 U/L)	298	71 (24.6%)	1.82 (1.15–2.90)	1.69 (1.05–2.71)	1.61 (0.99–2.61)
Q4 (>310 U/L)	262	108 (41.2%)	2.70 (1.72–4.24)	2.41 (1.52–3.84)	2.22 (1.37–3.58)
*P* for trend			0.000	0.000	0.000

^*1*^ Abbreviations: *GGT*, γ-Glutamyltransferase; *UA*, Uric acid; *IFG*, Impaired fasting glucose; *OR*, Odds ratio; *CI*, Confidence interval.

^*2*^ Multivariate logistic regression models were used to estimate the ORs and 95% CIs.

Model 1: adjusted for age, ethnicity, smoking, alcohol consumption, physical labor.

Model 2: Model 1 plus body mass index, waist circumference, systolic blood pressure, and diastolic blood pressure.

Model 3: Model 2 plus total cholesterol, triglyceride, high-density lipoprotein–cholesterol, and low-density lipoprotein–cholesterol.

## Discussion

In the present study, we compared the blood biochemical parameters between participants with prediabetes (i.e., IFG group) and those with normal glucose (i.e., NFG group) in a population derived from a cross-sectional survey. Logistic regression analysis showed that GGT and UA quartiles were positively correlated with IFG, and the associations remained after adjusting for possible confounding factors, including age, ethnicity, smoking, drinking, BMI, WC, BP, TC, TG, HDL-C, and LDL-C. To our knowledge, this was the first study to examine the possible associations of GGT and UA with prediabetes in areas inhabited by Chinese ethnic minorities.

Recent cross-sectional and longitudinal studies have found relatively independent associations between elevated serum GGT levels and hypertension or diabetes [18-21]. In the present study, among healthy men and women in Inner Mongolia, we found that higher serum GGT levels were positively associated with increased risk of prediabetes, suggesting that elevated GGT levels may provide early warning of diabetes, prompting interventions to prevent diabetes. Our results are consistent with those of previous studies examining the role of GGT in prediabetes [14,17,22]. In our study, we found that the OR (vs Quartile 1) for Quartile 3 was higher than that for Quartile 2, and was slightly higher than that for Quartile 4 (Table 3) in males, which suggests the effects of GGT on prediabetes outcome is non-linear among males. A similar pattern was observed for the association between GGT and peripheral arterial disease [23]. The possible reason for the non-linear association between GGT and IFG in this study is as follows: In adult males, increased GGT is often accompanied by dyslipidemia, probably because of the adverse eating habits (for example, people especially men from Inner Mongolia prefer drinking wine and eating meat). So the OR(vs Quartile 1) for Quartile 4 of serum GGT was slightly lower than that for Quartile 3 in Model 3 after ruling out lipid factors(including TC,TG,HDL-C,LDL-C), suggesting that the effect of lipids on FBG is the most obvious when GGT > 57 U/L.

At present, the underlying mechanisms for the increased risk of IFG in individuals with elevated GGT are unclear. In this study, BMI and WC were significantly greater in the IFG group than in the NFG group, suggesting that abdominal obesity may be involved. In fact, serum GGT levels are closely related to body fat [24]. Increased body fat promotes fat deposition in the liver and induces hepatic insulin resistance, thereby trigging systemic insulin resistance and the development of IFG. Another possible pathophysiological mechanism is that obesity is considered to be a chronic inflammatory state, and chronic inflammation induces the release abundant reactive oxygen species from mitochondria, resulting in oxidative stress. This promotes an increase in serum GGT, which has potential anti-oxidative stress properties, but ultimately damages the liver and other insulin-sensitive tissues [21,25].

It was suggested that UA may be a useful predictor of type 2 diabetes in older adults with IFG [26]. Recent studies have shown that patients with newly diagnosed type 2 diabetes have relatively low UA levels, but UA levels were significantly higher in people with IGT [16]. Moreover, in a 15-year follow-up study, Krishnan et al. reported that hyperuricemia in people aged in the mid-20s was an independent predictor of diabetes and prediabetes [15]. We found that women with IFG had lower UA levels than men, possibly because estrogen could promote urinary excretion of UA [27]. In addition, we found that women had higher OR values for IFG among each UA quartile than did men, after adjusting for confounding factors. Therefore, compared with men, women with high UA levels are at greater risk of prediabetes. Similar results were found in a cross-sectional survey of non-diabetic adults in Taiwan [28]. A recent study reported that UA is more strongly associated with impaired glucose regulation in women than in men [29], which further supports our conclusion.

Hyperinsulinemia is a marker of insulin resistance, a component of metabolic syndrome [30]. The relationship between UA and prediabetes may involve deposition of UA in islets, which could lead to β cell damage and functional decline, resulting in impaired glucose handling. The pro-inflammatory and oxidative stress effects of UA may also be possible causes of impaired glucose handling [31]. Several studies have suggested that serum leptin might be a pathogenic factor responsible for UA increase in obese patients [32-34]. Additionally, epidemiological studies have consistently shown that leptin is associated with glucose metabolism in patients with prediabetes [35,36]. Nevertheless, the reason why women with high UA levels are at greater risk of prediabetes than men is unclear and requires further clarification.

Some diets can have beneficial effects on uric acid level and fasting glucose. Dietary approaches to stop hypertension have beneficial effects on fasting glucose which can be related to improving some metabolic parameters linked to serum uric acid level and serum gamma-glutamyltransferase [37].

A major strength of our study is that it is a population-based study and the sample was a representative cohort of subjects living in Inner Mongolian Autonomous Region. Additionally, all data were collected using a standardized protocol with rigorous quality control. However, the study has several limitations to discuss. First, oral glucose tolerance tests were not performed, so we could not identify participants with IGT, another characteristic of prediabetes. It is possible that GGT and UA may show different associations with IGT than with IFG. Second, as we performed a cross-sectional survey, we could not determine causality or the temporal relationships among GGT, UA, and prediabetes.

## Conclusions

Our results demonstrate that GGT, UA, and IFG are closely associated in individuals located in areas inhabited by Chinese ethnic minorities. We found that higher serum GGT and UA levels were associated with greater risk of prediabetes, suggesting that they may serve as valuable clinical markers of prediabetes in China. Further studies are needed to elucidate the temporal nature of these associations, and to determine the reason why women with high UA levels are at greater risk of prediabetes than men.

## Competing interests

The authors declare that they have no competing interests.

## Authors’ contributions

JW carried out the experimental design, analysis, and interpretation of data, and drafted the manuscript. LQ conceived the study, participated in its design and coordination, and drafted the manuscript. WHY analyzed the data and drafted the manuscript. XQC and WW were responsible for data collection and revised the manuscript. XZG and HJH participated in the biochemical tests and revised the manuscript. HTD, SMH and GJZ participated in the design of the study and analyzed the data. All authors read and approved the final manuscript.

## Pre-publication history

The pre-publication history for this paper can be accessed here:

http://www.biomedcentral.com/1471-2458/13/294/prepub
